# New insights into the biology and ecology of *Acanthocheilonema reconditum *(spirurida: onchocercidae)

**DOI:** 10.1186/1756-3305-7-S1-O29

**Published:** 2014-04-01

**Authors:** E Napoli, G Gaglio, L Falsone, S Giannetto, F Dantas-Torres, D Otranto, E Brianti

**Affiliations:** 1 Dipartimento di Scienze Veterinarie, University of Messina, Messina, Italy; 2 Dipartimento di Medicina Veterinaria, University of Bari, Bari, Italy; 3 Department of Immunology, Aggeu Magalãhes Research Institute, Oswaldo Cruz Foundation, Recife, Brazil

## 

Among filarioids infesting dogs, *Acanthocheilonema reconditum *has a global distribution and epidemiological data indicate that it is the most prevalent or even the sole filarioid species-infecting dogs in some regions of the Mediterranean basin. For instance, in southern regions of Italy the prevalence is as high as 13.3%, and an annual incidence of 5.9% was estimated in naturally exposed dogs. In spite of its wide distribution and its suspected zoonotic potential, scant information is available on the biology of this filarioid. Nonetheless, recent studies have enhanced current scientific knowledge on the biology and ecology of this nematode in naturally infected dogs. Recent data indicate the absence of any defined periodicity of blood circulating microfilariae with peaks recorded either diurnal or nocturnal. With regard to the life cycle, fleas were confirmed to be vectors of *A. reconditum*, whereas the role of ixodid ticks (i.e., *Rhipicephalus sanguineus *sensu lato) as vectors of this filarioid species has been definitively rejected. The full development of microfilariae to infective forms occurs in the experimental infected cat flea *Ctenocephalides felis felis *in about 15 days. In addition, localization and size of developing larvae inside infected flea suggest that this arthropod might act as an intermediate host throughout the ingestion of infected fleas rather than the inoculation during the blood meal on dogs. If confirmed, this route of *A. reconditum *transmission is unique, differing from that of other filarioids affecting dogs such as *Dirofilaria immitis *and *Dirofilaria repens*, which are actively transmitted through the bites of mosquito vectors.

